# Contact to Nature Benefits Health: Mixed Effectiveness of Different Mechanisms

**DOI:** 10.3390/ijerph15010031

**Published:** 2017-12-25

**Authors:** Mathias Hofmann, Christopher Young, Tina M. Binz, Markus R. Baumgartner, Nicole Bauer

**Affiliations:** 1 Swiss Federal Institute for Forest, Snow and Landscape Research WSL, Economics and Social Sciences, Social Sciences in Landscape Research, 8903 Birmensdorf, Switzerland; christopher.young@wsl.ch (C.Y.); nicole.bauer@wsl.ch (N.B.); 2 Media Centre, Technische Universität Dresden, 01069 Dresden, Germany; 3 Center for Forensic Hair Analytics, University of Zürich, 8006 Zürich, Switzerland; tinamaria.binz@irm.uzh.ch (T.M.B.); markus.baumgartner@irm.uzh.ch (M.R.B.)

**Keywords:** chronic stress, hair cortisol, recreation, gardening, urban nature

## Abstract

How can urban nature contribute to the reduction of chronic stress? We twice measured the concentration of the “stress hormone” cortisol in the hair of 85 volunteer gardeners (six months apart), relating cortisol level change to (self-reported) characteristics of their recreational activities. Both time spent in nature and physical activity led to decreases in cortisol, while time spent being idle led to an increase. At high levels of present stressors, however, the relationship for time spent in nature and for idleness was reversed. Time spent with social interaction had no effect on cortisol levels. Our results indicate that physical activity is an effective means of mitigating the negative effects of chronic stress. The results regarding the time spent in nature and time spent being idle are less conclusive, suggesting the need for more research. We conclude that if chronic stress cannot be abolished by eradicating its sources, public health may take to measures to reduce it—providing urban nature being one effective possibility.

## 1. Introduction

### 1.1. Health and Stress

According to the World Health Organization, “health is a state of complete physical, mental and social well-being and not merely the absence of disease or infirmity” [1]. In this sense, health and well-being can be understood as synonymous. Instead of focusing on symptoms and causes of sickness, this understanding of health focuses on factors that constitute health in a holistic sense. The study described in this paper focuses on the factors contributing to human well-being and their interactions with the present levels of stressors.

Stressors are real or perceived threats to the perfect condition of the human body, known as homeostasis. Regardless of the specific nature of stressors, the human body exhibits a uniform stress reaction in their presence. This so-called fight-or-flight response is characterized by a rapid increase of physical readiness: to increase the supply of oxygen, energy and nutrients to the large muscles, blood pressure and heart rate are elevated, respiration intensifies, and fatty acids and glucose are released from reservoirs. Simultaneously, non-essential bodily functions are restrained, e.g., higher cognitive functioning, digestion, and immune reaction [2,3]. The stress response is subject to negative feedback processes, that is, it will normally be terminated when the stressor is no longer present. Responsible for this is a hormonal cascade involving the hypothalamic-pituitary-adrenal axis (HPA axis) during which the hormone cortisol is released [4].

Short-term stress may have positive consequences if the stress reaction facilitates successfully overcoming the respective stressor. Physical or psychological adaptation to a stressor will help to cope with this stressor in the future. This capability is referred to as allostasis, by which living organisms achieve stability by altering themselves, adapting to the allostatic load placed upon them by their environment [5,6]. If, however, inadequate coping with permanently present stressors leads to a continuous stress reaction (chronic stress), a number of negative physical and psychological consequences can be observed, such as anxiety disorders, sleeping problems, depression, cardiovascular problems, headaches, or abdominal pain [7,8].

In a poll among the Swiss working population, 17% of the respondents stated they suffer from work-related stress “always” or “most of the time”, with an additional 44% experiencing it “sometimes” [9]. Among German adults (18 to 79 years), 11% experience chronic stress, with women more often being affected than men (13.9% vs. 8.2%) and people of lower socio-economic status more often than people of higher socio-economic status (17.3% vs. 7.6%; [10]). Considering the negative effects of chronic stress on well-being, these numbers indicate that chronic stress constitutes a significant public health challenge.

Elevated levels of cortisol are often used as a physiological indicator of stress [4,11], with concentrations in saliva or blood being used to measure the short-term development of cortisol levels. The long-term development of cortisol levels can be seen as an indicator of chronic stress and may be assessed non-invasively by measuring the cortisol concentration in hair or fingernails [12,13,14]. As human hair on the head grows at a speed of approximately 1 cm per month, hair can be used to retrospectively measure cortisol levels for the preceding months. Hair cortisol concentration (HCC) has been previously used in studies such as a biomarker for major life stressors [15] or chronic stress [4] and also as indicator for the positive effects of vegetation on chronic stress [16,17].

### 1.2. Well-Being Effects of Contact to Nature

An increasing share of the population lives in urban environments [18] which traditionally feature comparatively few natural elements. At the same time, many stressors are present in these environments (e.g., noisy traffic, summer heat waves, spatial restrictions, high population density, and social isolation) with repercussions for human well-being [19,20]. While the notion of natural environments being beneficial to human well-being is very old [21], only in recent times this has been shown empirically. Numerous studies suggest that contact to nature has positive effects for human physical and psychological well-being (for overviews, see [22,23]).

City residents with more green spaces in their residential environments report better physical and psychological health than people from residential areas with fewer green spaces [24]. Taking a walk in a natural environment can improve subjective well-being [25]. Urban green increases the residential satisfaction [26] and life satisfaction in general [27]. Natural environments contribute to the restoration of attention after strenuous tasks (e.g., [28]), so that children with Attention Deficit/Hyperactivity Disorder (ADHD) who regularly play in green settings are better able to concentrate than comparable children who play in built settings [29]. Contact to nature can also strengthen human will power, leading to better and healthier lifestyle choices via an increased ability for delay of gratification [30,31]. It has also been shown that city residents deliberately engage in recreational activities in nearby nature to find restoration from work and personal life strains [32]. Exposure to natural environments can lead to lower blood pressure [33] and can even accelerate convalescence after surgery [34]. When studying the British population regarding the effects of income distribution on health, it was found that health-related differences between poorer and richer people are smaller in neighbourhoods with more green space [35], indicating that natural environments may be a means to reduce the effects of socio-economic status on health.

While benefits from contact to nature for human well-being have been frequently reported, it is not fully understood how these benefits come about. Possible mechanisms linking contact to nature to human health frequently have been discussed in the literature [36,37] among which are:

- improvement in micro-climatic conditions (e.g., air quality),
- stimulation of physical activity (e.g., exercise),
- facilitation of social cohesion, and,
- restoration from mental fatigue.

A number of previous studies researching these mechanisms used cross-sectional designs and collected data with questionnaires, thus relying on self-reports for the health outcomes. They found mixed results. While some studies reported no mediating effect of social contact or physical activity [38], others demonstrated that social support was a mediator between surrounding greenness and subjective general health, but physical activity only to a lesser extent [39]. Similarly, further studies found mediating effects of social cohesion and recovery from fatigue, but no clear mediating effect of physical activity [40].

Not only are the mechanisms of the nature–health relationship not fully understood and conclusively backed up by empirical research, it is also unclear whether the relationship between nature and health benefits is linear and which doses of nature exposure (at different levels) lead to which health responses [41].

### 1.3. Aims and Hypotheses

In the present study we aimed at identifying the relative role of the postulated mechanisms linking contact to nature to human health. We expected that each of the proposed mechanisms had a positive effect on human health. Thus, we hypothesized that in order to improve human health, recreational activities …

1. … taking place *in contact with nature* are more effective than indoor activities.
2. … involving *physical activity* are more effective than passive recreation.
3. … involving *social interaction* are more effective than those executed alone.
4. … allowing for *restoration from mental fatigue* are more effective than those that do not.

## 2. Methods

### 2.1. Study Overview

Recreational activities take place in different locations where different combinations of the proposed mechanisms may be present. Our study used urban gardening as an example of a recreational activity which potentially features all of these mechanisms simultaneously.

When participants choose their recreational activities themselves, the health effects may not solely be attributed to the chosen activity. Instead, certain demographic factors (or their interaction with the chosen activity) may be responsible for the health outcome. To reduce this self-selection bias, we recruited the participants from waiting lists for urban allotment gardens. All participants had applied for an allotment before the end of 2015. Because there were more applications than free allotments, it could be expected that not all participants would spend time in their own allotment garden during the 2016 gardening season. This meant that the other participants would have to choose different recreational activities, thus resulting in a sample with a quasi-random allocation to allotment holders and participants without allotments. However, we encountered two obstacles to this design. Firstly, people volunteering to participate in the study were mainly those who already knew they would receive an allotment. Secondly, most of those who did not receive an allotment managed to arrange gardening activities for themselves in other ways, such as gardening in friends’ gardens. This meant that almost the whole sample consisted of gardeners.

Despite lacking a non-treatment group, our sampling design does have certain advantages. We can expect participants to be similar in regard to dimensions we cannot measure well (or not at all) but which could have an influence on the relations between gardening and health outcomes. This is due to fact that all participants started gardening in the same season and of their own accord. One dimension where they should differ less than a heterogeneous sample of gardeners is their attitude towards gardening. Another such dimension is the experience of the first gardening year, where they can be expected to encounter similar delights and disappointments.

We collected data on recreational activities, present stressors, stress outcomes, and well-being status in spring (between mid-March and mid-May 2016; T1) and fall (between mid-September and mid-October 2016; T2) of 2016. In March 2016, the aspiring gardeners had received an invitation letter explaining the study. In follow-up phone calls, we provided them with further information on the study and set a date for a personal meeting, given they were prepared to participate in the study. At this meeting, we collected the questionnaire which had been sent along with the invitation. Also, we took a hair sample from each person which was later used to measure hair cortisol. This procedure was repeated in the fall with the same participants. All data were collected anonymously. The questionnaires and the hair samples were labelled with participant-generated codes to connect the data belonging to each participant.

The study was approved by the Cantonal Ethics Committee Zürich (Basec No. 2016-00057).

### 2.2. Participants

Study participants were recruited from allotment garden waiting lists in the Swiss cities of Bern, Basel and Schlieren. Of 710 aspiring gardeners having received a study invitation, a total of 140 persons participated in the study (response rate: 19.7%). Of these, 38 refrained from donating hair samples (questionnaire data only) and 32 dropped out before the second measurement. Excluding participants without hair samples and/or T1 data resulted in a sample of n = 85 study participants with complete data sets for both T1 and T2.

The median age of the participants was 38 years (range 25–70, mean 41, SD = 11.5; Swiss median age 42 years; [42]). Among the participants, 70% were female (Swiss population 50%; [42]), 40% were married or in a civil partnership (Swiss population 43%; [43]), 85.7% held Swiss citizenship (Swiss population: 76%; [44]), 14.4% lived in a single-person household (Swiss population: 16%; [45]), and 31.2% lived in a household together with children (Swiss population: 62.8%; [45]).

Regarding their highest educational qualification, 97.4% of the participants held a higher education entrance qualification or had completed vocational training and 78.2% held a university degree or equivalent (compared to 88.2% and 41.7%, respectively, of the Swiss population aged between 25 and 64 years; [46]).

In terms of occupation, 63.8% were employed, 16% were self-employed, 7.4% were in vocational training, 4.3% were unemployed and 8.5% were retired (Swiss population: employed 53.7%, self-employed 8.3%, training 7.5%, unemployed 2.9%, retired 21.1%; [47]).

Asked about the sufficiency of their monthly household income, 15% of the participants found it “difficult” or “very difficult” to get by with the available income (compared to 19.3% of the Swiss population whose satisfaction with their financial situation is low or very low; [48]).

In summary, comparing the participants to the Swiss population, notable differences concern gender (more women), the number of people living in households with children (fewer), the educational level (higher), and the share of pensioners (lower). The participants were rather similar to the Swiss population regarding age, marital status, employment, and satisfaction with household income.

### 2.3. Questionnaire

A printed questionnaire was sent to the potential subjects along with the invitation letter. Since all subjects lived in the German-speaking part of Switzerland, the questionnaire was administered in German. Besides demographic details, questions concerned recreational activities and exposure to nature at home and at work (for the items, see Section 3.2), and specifically the time spent in different types of gardens (allotment garden, private garden, community garden, other types of gardens). To answer these questions, participants were asked to indicate the average time per week, using a scale from 0 to 60 h which used 5-h intervals. (The participants could also indicate “more” hours per week. For the analysis, this was arbitrarily recoded to represent 80 h.) Although we assumed that recreational behaviour and exposure to nature at home and at work are habituated (and hence the answers can be expected to represent the whole time between T1 and T2), in the questionnaire the participants were asked to refer only to the last four weeks because we expected recollection accuracy to be higher for the last month than for the whole period.

The questionnaire also contained established scales which measured present stressors, observed stress outcomes, the current state of health, and current subjective well-being: present stressors were measured using the 12 item Screening Scale for Chronic Stress (SSCS) from a widely-used German stress inventory (TICS; [49]). Observed stress outcomes were measured using the mental and physical stress symptoms scale of the Stress and Coping Inventory (SCI; [50]). The current state of mental and physical health was assessed with the 12-Item Short-Form Health Survey (SF-12; [51]), using the established German translation [52]. To assess the current subjective well-being, the German short version (ASTS; [53]) of the Profile of Mood States (POMS; [54]) was administered.

We also asked the participants to indicate whether they had experienced a critical life event during the previous six months. Depending on its nature, such an event can be a major source of stress for multiple weeks or months, during which time it may effect perceived well-being and hair cortisol concentrations. We also asked for a short description of the respective event and interpreted these descriptions to identify positive events (e.g., engagement, wedding) negative events (e.g., serious illness, difficulties at work, unemployment, divorce, close relatives diagnosed with depression) and events which were neutral or potentially ambiguous (e.g., pregnancy, childbirth, retirement, start of studying for a second degree, changes in employment). We excluded three occurrences because we deemed them to be irrelevant or because they explicitly referred to events having occurred more than six months prior to T1 (e.g., global overpopulation, childbirth in September 2015).

### 2.4. Measurements of Hair Cortisol Concentration

We visited each participant who was willing to donate hair samples at a location convenient to them, often this was their home. At both T1 and T2, we obtained two hair samples per participant, each having a diameter of 2–3 mm. The strands were cut off from the posterior tip of the head, as close to the skull as possible. The hair samples were analysed in randomized order at the Center for Forensic Hair Analytics of the University of Zürich using a liquid chromatography–mass spectrometry (LC-MS/MS) method [55]. No other parameters were analyzed in the hair samples. For the statistical analyses, we used 3 cm hair segments closest to the head, roughly representing the three months previous to the time of the sampling.

### 2.5. Statistical Analyses

Where data points for single questionnaire items were missing from multi-item scales (e.g., SSCS), these were imputed using a hot-deck procedure with parametric mean matching (R package mice; [56]). This means that missing values were imputed on a single-item level, using the information of the items with non-missing data for each subject. Only afterwards were the items then further processed to calculate each subject’s value for a multi-item scale. Of the resulting imputed data sets, one was randomly selected and used for all further analyses.

To test the hypotheses, we used a multiple linear regression model because this allows for testing effects of the multiple predictors in interaction with each other and independently. The model predicts hair cortisol concentration (HCC) at T2, using the hair cortisol concentration at T1 as a fixed intercept. As regressors, we used the degrees to which each of the proposed nature–health-mechanisms were present in the recreational activities between T1 and T2 (e.g., hours per week spent with physical activity). The model can be conceptualized as follows: HCC_T2_ = 1 × HCC_T1_ + stressors × (mechanism_1_ + … + mechanism_4_). The four mechanisms corresponded to those in our hypotheses.

During regression diagnostics, we used visual inspections of the QQ plots and Cook’s D plots which can be used to identify overly influential observations that would threaten the stability of the model. Visually inspecting the plots, we excluded five outlier cases to gain a stable model. We also tested for multicollinearity among the predictors (to avoid instabilities of the regression model which may arise when variables are highly correlated) by calculating the condition index [57,58]. This yielded a maximum condition index of 22.48 which is well below the problematic rule-of-thumb threshold of 30 [58], indicating that multicollinearity is not a major concern for the present data. During inspection of the residual plots, we found no severe violations of the assumptions that the data be normally distributed (normality) and that the variances of all variables be homogeneous (homoscedasticity).

All statistical analyses were conducted using R (version 3.3.2, R Foundation for Statistical Computing, Wien, Austria; [59]), with the car package (version 2.1-4; [60]) and the perturb package (version 2.05) for regression diagnostics.

## 3. Results

### 3.1. Stressors, Well-Being Status, and Hair Cortisol Levels

The questionnaire contained several established scales to assess the participants’ well-being status and the stressors present in their lives. The descriptive statistics for these scales, as well as for hair cortisol levels, are presented in Table 1. There was no statistically significant change for any of the scales between T1 and T2. For some parameters, norm values for comparable populations were available, which were used for comparisons with the sample values at T2 (see Table 1). Compared to the norms, the participants were similar in terms of stress symptoms, but they reported more stressors, better physical health, and poorer psychological health. Although statistically significant, these effects are very small.

When asked for major life events during the previous six months at T1, 29 participants responded to have experienced one such event, with three of them being interpreted as positive events, 13 as negative, and 13 as neutral or ambiguous. At T2, 18 participants responded to have experienced one such event during the previous six months. Three of these events could be interpreted as a positive event, seven as negative, and eight as neutral or ambiguous.

### 3.2. Recreational Activities and Time Spent in Nature

In the questionnaire, the participants had provided information on the amount of time they spent with different recreational activities and in different natural environments (see Table 2). Although there were small differences between T1 (reported times referring to the first months of the year) and T2 (reported times referring to the summer), none of them were statistically significant.

To aggregate single activities or surroundings (e.g., time spent “… in the forest”, “… in urban nature”, “… in open landscape”, and “… at the water” to “… in natural environments”), we added up the single items in the respective category.

The participants also indicated how often they spent time in different types of gardens during the current gardening season. During the 2016 gardening season, 10% visited some type of garden on a daily basis, 48% multiple times per week, 24% multiple times per month, 8% multiple times per year, and 11% less often or never. The mean time which the participants spent in a garden during the gardening season was 17 h per week (SD 19, median 10). During this time, the mean amount of time they spent working there was 11 h per week (SD 17, median 5).

### 3.3. Correlations

Testing for correlations between the model variables we found that the level of stressors present was not correlated to any of the other variables (see Table 3). Among the other predictors, physical activity was significantly correlated to the other three predictors. Among them, no further correlations were significant. While the hair cortisol concentrations (HCC) at the two points in time (HCC_T1_ and HCC_T2_) were rather strongly correlated (ρ = 0.63), only HCC_T1_ was correlated to any of the other variables in the model; it showed a weak negative correlation with social interaction.

### 3.4. Regression Model for Hair Cortisol Prediction

**Figure 1:**
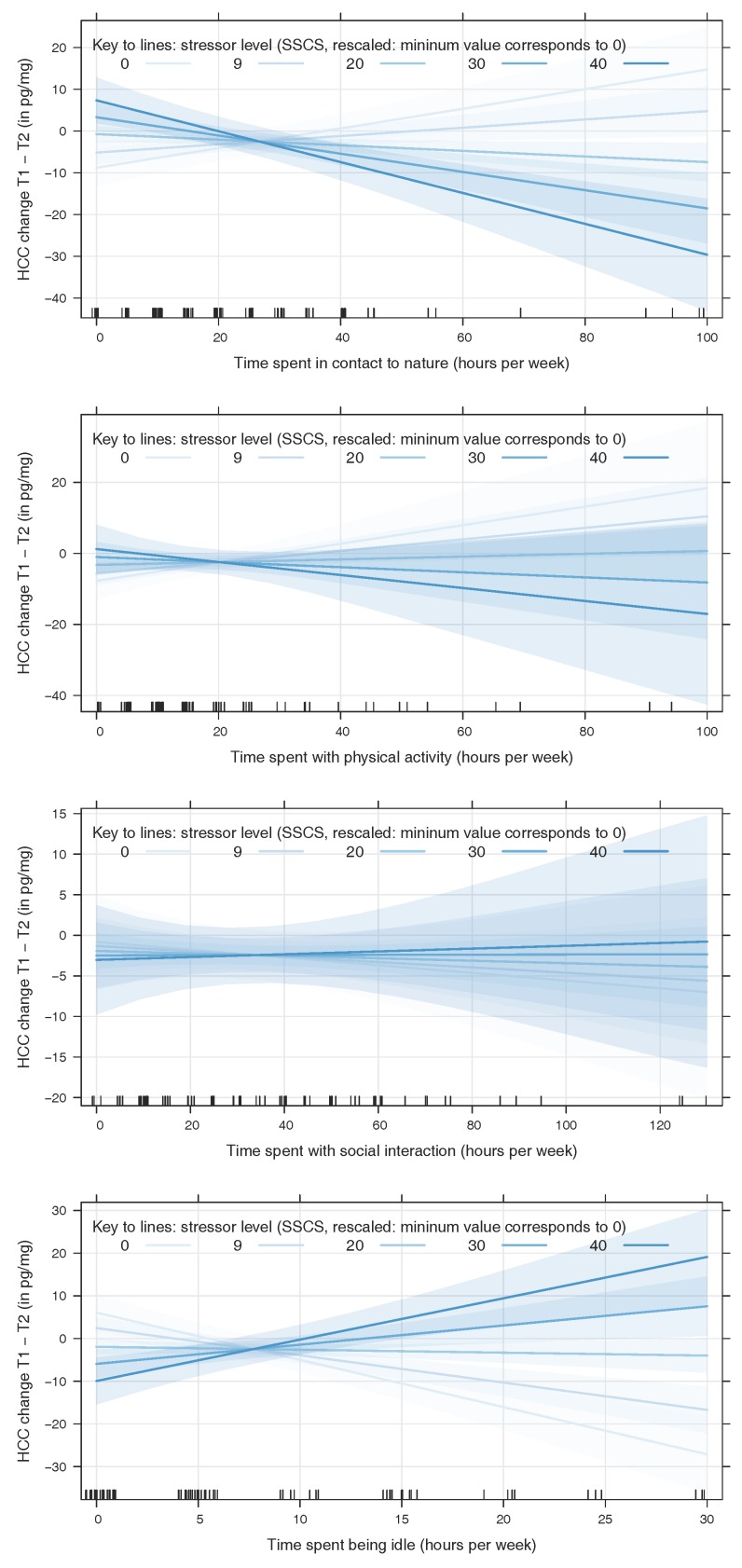
Interaction plots for the combined effects of stressor levels and characteristics of recreational activities during summer on the change in hair cortisol concentration (HCC, in pg/mg; differences between spring and fall measurements; higher values denote a more positive state, i.e., they correspond to a higher reduction of HCC). Only the interaction effects between time spent in nature and stressor level (upper panel) and between idleness and stressor level (lower panel) were statistically significant (see Table 4).

We tested the theoretically derived regression model using the collected data. Of the four mechanisms, three were statistically significant predictors of HCC: while increases in the time spent with recreational activities involving nature contact (time spent in natural environments, see Table 4) and physical activity led to a decrease in HCC in our model, increases in time spent being idle let to an increase in HCC. The present stressors on their own had no significant effect. However, they were important in interaction with two of the mechanisms in that they reversed their effect on HCC (see Table 4 and Figure 1).

To check the stability of the model, we generated multiple partial models by systematically excluding all possible different combinations of the predictors. While the magnitudes of the predictors varied during this procedure, their signs remained unchanged, indicating overall stability of the model.

Another check on the stability of the model was conducted using ordinary non-parametric bootstrapping (using 1000 repetitions). This yielded similar values for the determination coefficients: *R*^2^ = 0.3686 (bias = 0.0545, *SE* = 0.1320, 95% CI: 0.1424–0.5944), adj. *R*^2^ = 0.2874 (bias = 0.0696, *SE* = 0.1404, 95% CI: 0.0089–0.5158).

When demographic variables (e.g., gender, age, marital status, or employment) were added to the model as predictors, they did not have a significant effect. Similarly, adding interaction terms allowing for interactions between each two of the predictors neither altered the model significantly nor did it yield additional significant interaction terms.

We also tested the model by systematically excluding each and all subgroups of participants who had indicated different types of major life events at T1 or T2 (positive, negative, or neutral/ambiguous). This did not lead to changes in the signs or magnitudes of the model’s beta weights; only to a loss of statistical significance for physical activity when all participants with major life events were excluded. An alternative strategy (dummy-coding the different types of major life events and including them as additional predictors) also did not change the model: none of these additional variables had significant beta weights. Also, the signs, magnitudes, and significance levels of the original model’s beta weights remained unaltered.

## 4. Discussion

To determine the relative importance of different mechanisms linking nature to human health, we tested a theoretically derived regression model which used the level of self-reported stressors in interaction with weekly durations of contact to nature (time spent in natural environments), physical activity, social interaction, and idleness to predict hair cortisol concentration (HCC), the latter serving as biomarker for chronic stress. We found that the model could explain changes in HCC rather well. Among the predictors, nature contact, physical activity, and idleness influenced HCC, while no effect of social interaction was present.

In the present study, we found no statistically significant differences in HCC between the spring (T1) and fall (T2) measurements. Seasonal effects and effects of duration of sample storage on measured HCC have been reported in previous research [61,62,63]. According to these studies, one would expect lower HCC for the summer season (which we measured at T2) and lower values for the samples that had been stored for longer (which concerns all samples taken at T1, because they were analysed only after T2). However, for the purposes of this study, this does not pose a problem, because the seasonal effects were equal for all study participants, irrespective of stressor levels or recreational behaviors. Possible HCC differences due to storage duration are also unproblematic for the analysis because storage duration for all samples taken at T1 was approximately equal for all subjects.

### 4.1. Contact to Nature and Physical Activity Mitigate Stress While Idleness Intensifies Stress Outcomes

In the regression model, only nature contact and physical activity have beneficial effects on HCC on their own, in that increases in either lead to decreases in HCC. This lends support to our hypotheses 1 and 2 (however, in case of hypothesis 1 this support is limited by the levels of present stressors; see below). Also, this finding is in line with previous studies on the well-being effects of nature contact [23].

In contrast to nature contact (see below), we found no interaction effect for levels of present stressor with physical activity, indicating that physical activity may be beneficial irrespective of stressor levels. The relation of our findings regarding physical activity to previous studies is however less clear-cut because some of these studies found physical activity to be of greater importance in mediating well-being effects (e.g., [25]) whereas others found physical activity to be less effective (e.g., [39,40]). Seeing different results in different studies is of course not unusual and may be caused by differences in these studies regarding their design or the way they measure the environmental characteristics, the physical activities, or the well-being outcomes.

Idleness has an overall negative effect on HCC, meaning that more time spent being idle is generally associated with higher HCC. A possible interpretation of this effect could be that because time is a limited resource, time which a person spends being idle generally means less time during which health benefits from contact to nature or physical activity can be generated. One might argue that time being idle may be spent in contact to nature, but the low correlations between the two parameters (see Table 3) suggest that this was not the case in the present study. Another possible explanation for the negative overall effect of idleness on HCC is that being idle may mean that certain stressors are allowed to persist longer. Choosing to put off stressors which can only be dealt with through some form of personal effort (instead of being idle) can result in mood improvements in the short term but may also lead to prolonging or increasing the impact of the ignored stressor, and have negative consequences for psychological well-being in the long term [64].

### 4.2. Inverse Effects of Nature Contact and Idleness at High Levels of Stress

At higher levels of present stressors, this picture is altered considerably: Additional hours spent in contact with nature lead to higher HCC. Hence, while nature contact has a stress-reducing effect for participants with low stressor levels, this effect is inverse when stressor load is high. This considerably restricts the support of the present data for our first hypothesis. This could be a result of trying to purposely mitigate chronic stress by following a popular advice (“spend time in nature”) which may create even more stress by demanding additional time on an already filled agenda.

When high levels of stressors are present, the effect of idleness is also reversed; increases in idleness then go along with decreases in HCC. This represents partial support for our fourth hypothesis insofar as one might consider being idle as contributing to restoration from mental fatigue. It may be speculated that taking time to be idle *when* already stressed could help reduce chronic stress because it allows for time to calm down, to reflect on the stressors and possibly to find solutions to problems.

### 4.3. No Effects of Social Interaction

Social interaction did not influence stress outcomes, neither directly nor in interaction with stressor levels, hence the present data do not support our third hypothesis. This is especially remarkable because compared to the other recreational activities, social interaction was the activity that the participants spent the most time with. Because the variance for this activity was highest compared to all other recreational activities, the lack of effect on HCC is unlikely to be caused merely by a lack of variability within the activity.

This is in contrast to previous research where social support has been found as a protective factor in coping with stress [65,66,67,68] and as beneficial to different aspects of health, such as cardiovascular, neuroendocrine, and immune function [69]. These studies however concern the effects of stressful life events and how they may be mitigated via social support, whereas in the present study we tried to focus on the effects of *chronic* stress. While social support may be an important protective factor in coping with severe, acute stress events, it may be less important for dealing with more low-key, “everyday” stress.

Furthermore, in contrast to the mentioned studies, we did not measure the extent of social support, but the time spent with interacting socially, that is, time spent with family and friends. Although spending time with potentially helpful others certainly is a prerequisite for social support, it may not be sufficient to show a significant effect. We also did not collect data on personality traits which may explain possible individual differences, i.e. that social interaction may be more or less desirable, and may accordingly have different outcomes, depending on where a person stands on the extroversion–introversion continuum.

Also, time spent with other people itself may even be a source of stress when there are conflicts with these people or when they themselves face severe illness of other problematic situations in life (e.g., [70,71,72]). Although one may be able to reduce the contact to acquaintances if this proves a burden to oneself, this may be infeasible with family members of spouses. However, in studies researching the connection between urban greenness and self-reported health [39,40] social support has been shown to be a mediator.

### 4.4. No Main Effect of Present Stressors

Although the present stressors were central to the regression model in that they influenced the effects of nature contact and idleness on HCC, there was no measurable main effect of the stressors on this stress outcome. Also, there were no correlations of present stressors and HCC. Although we did not hypothesize any main effect of the present stressors on HCC, one might intuitively expect such an effect. A possible explanation for the lack of this effect may be grounded in the characteristics of the sample. Because we wanted to limit possible distractive effects of sociodemographic factors on stress outcomes, we tried to recruit participants which were rather homogeneous regarding their sociodemographic background. This may have led to fewer variations in the present stressor levels. Compared to the SSCS questionnaire norm where the SD is 10 points, we observed a lower SD for our sample (7.8 points). The same be the case for stress outcomes, in that small variation in stressors may have led to smaller variations in stress outcomes. Together with a rather small sample size, these lacks of variation may have prevented finding a robust main effect.

### 4.5. Representativeness of the Participants

The characteristics of the participants in the study sample suggest that the relations our results display should hold for relatively healthy populations with an intent to garden resident in urban areas, at least in wealthy Western European countries.

The targeted sample of 710 aspiring gardeners consisted of persons resident in urban areas of German-speaking Switzerland with an intention (validated by the fact they had taken steps to get an allotment garden) to engage in gardening as a leisure activity. While this is not a random selection of cities or gardeners within cities, there are no apparent theoretical reasons why the relations found in our analysis should not hold for persons with an intent and the (financial) means to garden in other Swiss or Western European cities. The demographic structure of the study sample of 85 participants is similar to the general Swiss population in regard to age, civil status and their subjective evaluation of their household income. The over-representation of women (70% vs. 50% in the population) may partly be due to the fact that women are generally more likely to be involved in gardening than men. The Swiss time use study suggests this, as the average time per week spent tending to gardens, plants or pets is higher for women than for men [73]. Thus, the sample may represent the population of people with intent to garden correctly in respect to gender. The over-representation of people holding a higher education degree is perhaps slightly more critical. General observations in Switzerland suggest that allotment gardening has substantially gained in popularity among people with a high level of educational attainment. Thus, our sample (which consisted of people newly engaged in gardening) may merely reflect this trend.

A characteristic of the sample which may prevent us from identifying effects is the generally moderate level of stress. Because the study participants had applied for an allotment garden and in addition were willing to partake in a scientific study, one may assume that they would at least have enough time for these activities. Stress levels may have been higher in persons who had too little time for participating, e.g., due to higher workloads or more extensive family obligations. In a sample with higher stress levels however, factors which show no relation to stress reduction in our model may well have an effect. As the level of stress is higher, stress reducing factors have more scope to take effect. The absence of a significant relation of social interaction to stress reduction may be due to this.

### 4.6. Limitations and Consequences for Future Research

Our study illustrates to some extent the difficulties of designing quasi-experimental research to examine the effects of nature exposure over long time periods (i.e., over months). Such designs require a treatment group and a control group, (close to) random allocation of participants to one of the groups and clear distinction between the groups. In our case, allocation of potential participants (i.e., persons on the waiting lists) to an allotment was not fully random, but probably not systematically related to the relevant factors in our model. However, the long duration of the study meant that participants who did not receive an allotment could find other opportunities to engage in gardening, thus reducing the potential effects of gardening.

In our case, however, another issue had graver consequences. Very few persons who had not received an allotment participated in the study. This was due to the fact that during our recruiting phase, many aspiring gardeners already knew whether they would receive an allotment for the coming spring or not. Given a focus on gardening in the recruiting documents, people who had not received a garden apparently did not feel the study regarded them. Future studies looking to recruit groups of gardeners and non-gardeners would be advised to frame the research as pertaining to leisure in general and not to gardening specifically.

In sum, this led us to abandon an initially intended quasi-experimental design and to attempt to identify relations between independent and dependent variables with cross-sectional regression methods. In contrast to a standard cross-sectional design with only one measurement point, the two measurement time points make it possible to identify changes over time on the level of the individual and relate it to certain factors.

Thus, the results of our study must be considered in light of certain limitations. The moderate stress level of participants mentioned above means that some aspects of gardening may contribute to stress reduction for persons experiencing more stressors, even though these aspects do not appear as significant effects in our data. Furthermore, given the design issues discussed above, the variation of time spent gardening was limited in our sample, particularly as there were almost no real non-gardeners. This could also lead to an underestimation of the effects of gardening on cortisol levels and stress in our sample.

Finally, we used a study design in which the data on the characteristics of the recreational activities was collected using participants’ self-reports. Asking participants to recall information is prone to flaws in human memory which may have altered the observed effects. Although we tried to minimize such biases, for example by asking about short periods of time in the past, future research may consider using direct observation or successive questionnaires each pertaining to shorter periods of time.

## 5. Conclusions

The present study supports the notion of natural environments being beneficial to human well-being. Given the current and expected rises in both population and urbanization, it seems important to sustain and extend urban green spaces in order to provide recreational spaces for increasing numbers of city residents. Considering the magnitude of the effects in our study, it seems appropriate to prioritize abolishing the sources of chronic stress rather than merely trying to remedy its negative consequences to human health. Nonetheless, our results emphasize the importance of physical activity for human well-being, irrespective of stressor levels.

Certainly not the whole population will be willing or able to take on gardening, so the conclusions for public health from a “gardening study” may be seen as limited in this regard. However, we used gardening as model activity, which to different extents features the mechanisms which are of interest for studying the benefits from nature to well-being. Hence, other recreational activities also featuring these mechanisms may be similarly effective.

Urban planning may hence try to facilitate different kinds of physical activities in natural environments for city residents. These activities may be of very different kinds and be afforded by very different design decisions, depending on the local situations. For example, urban gardening may be promoted to facilitate physical activity in urban nature settings. Also, it might be desirable to make inner-city areas more accessible and attractive to pedestrians in order to facilitate walking, or to establish phased traffic lights adjusted to typical biking speeds or “green corridors” which make cycling more attractive in daily commutes. In general, if public health policies regarding chronic stress are to be translated into actual urban planning decisions, it seems advisable to aim for measures which afford physical activity and contact to nature in ways which can be easily incorporated into stressful daily lives without much effort.

## Figures and Tables

**Table 1:** Descriptive statistics for the scales used in the questionnaires and the cortisol measurements.

Parameter		T1			T2		M_T1_ = M_T2_	Norm		M_T2_ = M_Norm_
	MD	M	SD	MD	M	SD	t_DF_^p^	M	SD	t_DF_^p^
Stressors (SSCS) ^*inv.*^	54	54.2	8.1	52	52.5	7.8	1.32_157.8_	50	10	2.9_79_ **
Stress symptoms (from SCI) ^*inv.*^	36	36.4	6.5	34	35	5.6	1.43_154.9_	34	n/a	1.6_79_
Physical health (SF12)	52.8	49.9	8.7	54.2	52.1	6.2	−1.87_143.5_	49.03	9.35	4.4_79_ ***
Mental health (SF12)	51.1	49.1	8.8	52	49.8	7.3	−0.55_152.8_	52.24	8.1	−2.9_79_ **
Mood (ASTS) ^*inv.*^	0	1.2	15.9	−4	−2.5	13.2	1.61_152.7_	n/a	n/a	
HCC (pg/mg) ^*inv.*^	6.2	8.5	10.3	7.6	10.8	11.2	−1.35_156.8_	n/a	n/a	

Note. “*inv.*” indicates that smaller values denote “better” states, i.e., fewer present stressors, fewer stress symptoms, better mood, and lower cortisol levels; HCC: hair cortisol concentration; T1: spring 2016; T2: fall 2016; results of test for differences between these two values in column “M_T1_ = M_T2_”; MD: median, M: mean; SD: standard deviation; sig. levels: ** *p* < 0.01; *** *p* < 0.001; n/a: data not available.

**Table 2:** Time spent with recreational activities and in natural environments. None of the differences between T1 and T2 were statistically significant.

Activity		T1			T2	
Time (hours/week) spent …	MD	M	SD	MD	M	SD
… just being there, doing nothing (idleness)	5	6.9	10	5	7.8	8.6
*… with physical activity (combined)*	12.5	18.2	19	15	19.7	18.6
… with light physical activity	10	13.2	15.1	10	12.4	13
… with intense physical activity	5	5	9	5	7.3	10.9
*… with social interaction (combined)*	20	35.8	32	30	37.1	29.2
… with friends	10	15.9	17	10	16.6	16.7
… with family	10	19.9	19.7	15	20.6	19.6
*… in natural environments (combined)*	15	23.4	25.2	20	26.3	22.9
… in the forest	5	3.8	4.4	5	3.9	4.5
… in urban nature (e.g., in a garden)	5	9.1	10.3	5	8.8	9.2
… in open landscape	5	5	7.4	5	5.9	7.4
… at the water	5	5.6	8.7	5	8.4	11.4
… outdoors (other environments)	5	12.6	15.1	10	13.2	13.2

Note. Italicized activities represent means of the indented activities in the rows below them. T1: spring 2016; T2: fall 2016; MD: median, M: mean; SD: standard deviation.

**Table 3:** Intercorrelations (Spearman’s ρ) of the predictors and correlations with hair cortisol levels.

	Idleness	Nature Contact	Social Interaction	Physical Activity	HCC_T1_	HCC_T2_
Stressors (SSCS)	−0.03	0.03	−0.09	−0.05	0.06	0.03
Idleness		0.16	0.13	0.37 ***	−0.04	0.15
Nature contact			0.19	0.36 ***	0.16	0.12
Social interaction				0.44 ***	−0.23 *	−0.1
Physical activity					0.01	0.11
HCC_T1_						0.63 ***

Note. HCC: hair cortisol concentration. Sig. levels: * *p* < 0.05; *** *p* < 0.001.

**Table 4:** Regression model predicting hair cortisol concentration (HCC) at T2 using stressors (SSCS) and attributes of recreational activities as predictors and HCC at T1 as a fixed intercept. *R*^2^ = 0.3686, adj. *R*^2^ = 0.2875, F_9,70_ = 4.541, *p* < 0.001.

	β	*SE*	*t*	*p*
Intercept	−3.5191	2.6935	−1.3065	0.1957
Stressors	0.1565	0.1337	1.1706	0.2457
Nature contact	0.2354	0.0656	3.5894	0.0006 ***
Physical activity	0.2612	0.1154	2.2623	0.0268 *
Social interaction	−0.0476	0.0687	−0.6928	0.4907
Idleness	−1.1058	0.1974	−5.6018	0.0000 ***
Stressors × Nature contact	−0.0151	0.0035	−4.2682	0.0001 ***
Stressors × Physical activity	−0.0111	0.0064	−1.7389	0.0864
Stressors × Social interaction	0.0016	0.0035	0.4602	0.6468
Stressors × Idleness	0.0519	0.0104	4.9776	0.0000 ***

Note. Sig. levels: * *p* < 0.05; *** *p* < 0.001.
